# Changes in family knowledge, practices, and perceived competencies after a family-centered home-visiting program for chronic child malnutrition prevention in rural Ecuador: an uncontrolled pre-post study

**DOI:** 10.3389/fpubh.2026.1925365

**Published:** 2026-10-26

**Authors:** Claudia Tatiana Escorcia-Mora, Marcela Frugone-Jaramillo, Pau García-Grau, Carolina Portaluppi, Diana Barros-Cárdenas, María Alejandra Muñoz-Seminario

**Affiliations:** 1 Campus Capacitas, Catholic University of Valencia San Vicente Mártir, Valencia, Spain; 2 General Directorate of Research, Innovation and Creation, Universidad Casa Grande, Guayaquil, Ecuador; 3 Graduate School, Universidad Casa Grande, Guayaquil, Ecuador; 4 Universidad Casa Grande, Guayaquil, Ecuador; 5 Social Investment and Development of the Guayaquil Charity Board, Guayaquil, Ecuador

**Keywords:** chronic child malnutrition, community health, early childhood intervention, family support, Family-Centered Practices, parenting competencies, prevention

## Abstract

**Introduction:**

Chronic child malnutrition (CCM) remains a major public health challenge, particularly in socially vulnerable settings, given its association with adverse developmental outcomes. Preventing CCM requires integrated approaches that combine nutritional support, healthcare, and the strengthening of family caregiving capacities.

**Objective:**

To describe pre-to-post changes over 6 months in parental knowledge (primary outcome) and in practices and perceived competencies (secondary outcomes) related to the prevention and management of CCM among 208 pregnant women or mothers of children under 3 years of age participating in a family-support process guided by the principles of Family-Centered Practices (FCP) in a rural community in Santa Elena, Ecuador.

**Methods:**

An uncontrolled longitudinal pre-post study with an embedded exploratory qualitative component was conducted within a community program involving biweekly home visits and monthly food kits. No concurrent comparison group was used. A 34-item, study-specific scale assessing knowledge, practices, and perceived competencies related to CCM prevention and management was administered before and after the intervention. In addition, 25 purposively selected mothers provided written responses to open-ended questions, which were analyzed deductively using four *a priori* categories.

**Results:**

The total scale score increased significantly from pre- to post-assessment (mean difference = 8.03, 95% CI [4.53, 11.53], *p* < 0.001, *d*z = 0.31). The largest and only statistically significant domain-level change was observed in Knowledge (*d*z = 0.66), whereas changes in Practices (*p* = 0.425) and Competencies (*p* = 0.080) were smaller and not statistically significant. Sixteen of 34 item-level comparisons remained significant after Holm-Bonferroni correction. Differences according to baseline nutritional status were descriptive and were not confirmed by the group-by-time interaction test. Qualitative responses illustrated perceived learning related to safe water, dietary practices, and greater confidence in food-related decision-making.

**Conclusion:**

Participation in an integrated home-visiting and food-support program was associated with higher self-reported knowledge related to CCM prevention and management, whereas aggregate changes in practices and perceived competencies were not statistically significant. Given the uncontrolled design, concurrent food support, and use of a study-specific outcome measure, the findings should be considered preliminary. Controlled studies using validated measures and objective nutritional outcomes are needed to evaluate the contribution of family-centered support to CCM prevention.

## Introduction

Chronic child malnutrition (CCM) remains one of the most persistent public health challenges in Latin America. Commonly reflected in stunting, it is associated with long-term adverse effects on physical, cognitive, and social development, particularly when it occurs during the early years of life (1–4). In socially vulnerable contexts, CCM may contribute to developmental trajectories characterized by reduced opportunities for learning, autonomy, and social participation, highlighting the importance of early preventive interventions.

Although stunting is the primary anthropometric indicator of chronic malnutrition, other indicators of child undernutrition, such as underweight and wasting, provide complementary information on children’s nutritional status and should also be considered when describing the broader nutritional context. Recent evidence also indicates important relationships between wasting and stunting across early childhood, supporting the need to consider different manifestations of undernutrition within an integrated framework (5).

In Ecuador, the most recent national survey specifically focused on child malnutrition (ENDI 2022–2023) estimated the prevalence of stunting at 20.1% among children under 2 years of age and 17.5% among children under 5 (6). Prevalence is markedly higher in the province of Santa Elena, where the present study was conducted: 29.8% among children under 2 years, representing the third-highest prevalence among the country’s 24 provinces, and 27.5% among children under 5 (6). As ENDI reports data specifically on stunting, information on other anthropometric indicators was obtained from the most recent national health and nutrition survey (ENSANUT 2018), which does not provide province-level estimates for these indicators. At the national level, wasting affected 3.7% (95% CI: 3.2–4.2) and underweight 5.2% (95% CI: 4.6–5.7) of children under 5 (7).

CCM cannot be understood or addressed solely as a nutritional or health problem. Its emergence and persistence result from the interaction of multiple structural, community, and family-level factors, including poverty, limited access to safe water and sanitation, inadequate access to healthcare services, maternal education, food insecurity, and everyday feeding and caregiving practices (8–12). This burden is also unevenly distributed across the Ecuadorian population, disproportionately affecting children living in rural areas and communities experiencing long-standing social disadvantage (6, 11, 13). Evidence from Ecuador also indicates that feeding practices, social and community resources, and broader household circumstances are relevant to children’s nutritional status (13, 14).

From an ecological perspective on human development, families play a central role in shaping the routines, interactions, and caregiving practices that influence children’s well-being and development (15). Recognizing this role does not imply assigning responsibility for developmental outcomes solely to families. Rather, it acknowledges that, particularly in contexts of social inequality, families require respectful, sustained, and context-sensitive support to strengthen their caregiving capacities (16, 17).

Although research has consistently shown that parental knowledge regarding nutrition, hygiene, and healthcare is relevant to the prevention of CCM, knowledge alone is rarely sufficient to produce sustained changes in everyday practices, which also depend on household resources, food availability, cultural norms, decision-making patterns, and access to healthcare, water, and sanitation (8, 9, 18, 19). Evidence from behavior-change and family-engagement interventions also indicates that improving nutritional practices requires more than providing information alone and that family and contextual conditions influence how recommendations are incorporated into everyday life (19, 20). Support processes that combine information, reflection, and collaborative decision-making within real-life situations may therefore facilitate gradual and contextually meaningful changes in family routines and caregiving practices (17, 21, 22).

Within this framework, Family-Centered Practices (FCP) provide a particularly relevant approach. FCP emphasize collaborative partnerships between professionals and caregivers, build on family strengths and priorities, and promote shared decision-making and support embedded within everyday routines and natural environments (23–26). Contemporary evidence continues to support the relevance of relational and participatory processes within family-centered intervention and their association with outcomes such as family empowerment, parenting capacity, achievement of family goals, and satisfaction with services (17). Research has also highlighted the relevance of family confidence and perceived competence within family-centered early intervention (16, 27). Within this study, competencies are understood as parents’ perceived capacity to make decisions, solve problems, and respond effectively to situations related to child nutrition and caregiving.

Although the FCP approach was originally developed within early intervention services for children with disabilities or developmental-risk conditions, where substantial empirical support has accumulated (17, 22, 28), its underlying principles are applicable to broader contexts in which families require support to sustain caregiving and child-rearing practices. Of particular relevance to the present study, Family-Centered Practices have already been implemented with families living in rural and socially vulnerable contexts in Ecuador, with reported changes in family quality of life, child development, and families’ self-perceived competence (16). Extending the FCP approach specifically to the prevention and management of chronic child malnutrition represents a distinct application, for which empirical evidence remains limited.

Home visits constitute an established strategy for delivering family support in community settings. Their effectiveness depends on several factors, including the quality and consistency of the intervention, the training and support of community workers, and the extent to which intervention goals are integrated into families’ everyday routines (21, 22, 29, 30). In rural Peru, Araujo et al. (21) evaluated the large-scale Cuna Más home-visiting program and found modest but significant improvements in child development as well as changes in selected parenting behaviors. These findings provide contemporary Latin American evidence for the potential of structured home visiting delivered through community services, although the objectives and intervention content differ from those of the present study. Despite this evidence, empirical research specifically examining family-support models based on FCP for the prevention and management of CCM in rural Latin American communities remains limited.

The present study was carried out within a community-based program for the prevention and reduction of chronic child malnutrition implemented in a rural municipality in the province of Santa Elena, Ecuador. Its aim was to describe pre-post changes over 6 months of family-centered support in parental knowledge (primary outcome) and in practices and perceived competencies related to CCM prevention and management (secondary outcomes), and to explore, on an exploratory, non-pre-specified basis, whether these changes differed by the family’s baseline nutritional-risk status.

## Method

An uncontrolled, longitudinal, descriptive pre-post design was used, with an embedded, exploratory qualitative component. The quantitative component examined changes in scores on a scale of parental knowledge, practices and competencies. Written responses to open-ended questions about perceived learning and evaluation of the support were collected as part of the qualitative component, which was of an exploratory nature. All families enrolled in the program’s first cohort completed both assessments. *Post hoc*, the study had approximately 80% power to detect dz ≥ 0.20 (α = 0.05, two-tailed) for the total-score comparison, but less power for the smallest nutritional-status subgroup (*n* = 25).

### Context and participants

The research was conducted in a rural community in the province of Santa Elena, Ecuador, which is characterized by conditions of social vulnerability and a high local prevalence of chronic child malnutrition (CCM). The study was part of a program for the prevention and eradication of chronic child malnutrition that was promoted and funded by the Guayaquil Charity Board, in coordination with community stakeholders and local public services, and with academic support from Universidad Casa Grande.

The program conducted an initial screening through a community census to identify families with pregnant mothers or children up to 3 years of age. Families residing in the community who expressed a willingness to participate were included, regardless of whether the child had been diagnosed with CCM with no additional exclusion criteria beyond non-residence or unwillingness to participate; one participant (the mother or, in her absence, the primary caregiver) was enrolled per household. Participants aged 18 years or older provided informed consent directly; for participants under 18 (*n* = 8 in the analytic sample), written assent was obtained from the adolescent together with informed consent from a parent or legal guardian, following the protocol approved by the institutional review board. Eligibility for food kits and other community services was independent of participation in this research component.

A total of 208 mothers (*n* = 208) who enrolled at the start of the first intervention semester and completed the data collection procedures were included in the analysis. Fifty mothers who joined the project after the first pre-assessment window had closed were excluded, since they had no Pre-FCP measurement to pair with a Post-FCP one; this exclusion was decided prospectively, before any outcome analysis. We could not formally compare these families with the analyzed sample on baseline characteristics, and if later enrollment correlated with family characteristics such as mobility or engagement, the analyzed sample may not be fully representative of the total population served by the program; this is noted as a limitation. The mean age of the participants was 29.13 years (SD = 6.45), ranging from 13 to 45 years. The mean age of their children was 14 months. 18.26% of the participants were pregnant at the time of the study.

Regarding the nutritional status reported in the household, 55.8% indicated that they had no children with anemia or CCM. Among the remaining 43.7%, 12.0% reported anemia, 19.2% reported anemia and CCM, and 12.5% reported CCM, while 0.5% did not provide this information. Nutritional status was based on caregiver report of a prior clinical diagnosis or screening result communicated by a health provider, rather than on anthropometric or biochemical measurement performed by the research team (e.g., WHO Anthro, MUAC, or hemoglobin testing) and should be interpreted as indicative rather than diagnostic. See Table 1 shows the full baseline-characteristics description (maternal age, pregnancy status, presence of an additional adult caregiver in the household, and child age); maternal education, household income, parity, ethnicity, and water/sanitation access were not systematically captured by the intake instrument used for this cohort.

**Table 1:** Baseline characteristics of the analytic sample (*N* = 208).

Characteristic	Value
Maternal age, years, M (SD) [range]	29.13 (6.91) [13–45]
Participants younger than 18 years, *n* (%)	8 (3.8%)
Pregnant at baseline, *n* (%)	38 (18.3%)
Additional adult caregiver in the household, *n* (%)	187 (89.9%)
Child age, months, M (SD)	14.0 (9.2)
Nutritional status (caregiver-reported)	
No anemia or CCM, *n* (%)	116 (55.8%)
Anemia only, *n* (%)	25 (12.0%)
Chronic child malnutrition (CCM) only, *n* (%)	26 (12.5%)
Anemia + CCM, *n* (%)	40 (19.2%)
Not reported, *n* (%)	1 (0.5%)

*N* = 208. Maternal education, household income, parity, ethnicity, and water/sanitation access were not systematically captured by the instrument and could not be reported.

#### Description of the intervention

The intervention consisted of a family support process delivered through biweekly home visits over a six-month period. It was implemented as part of a broader community program that also included the monthly distribution of food kits. Consequently, the interpretation of the observed outcomes should take into account that family support was not the only component received by participating families.

All enrolled families received the same monthly food kit, independent of participation in the FCP home-visiting component. Eligibility for the kit was based on enrollment in the program, and all enrolled households received approximately 20 kg of food per month. According to program records, the kit included cereals and derivatives (rice, pasta, and oats), dried legumes (beans, lentils, and chickpeas), canned fish (tuna and sardines), powdered milk, vegetable oil, brown sugar, chocolate, and salt. The kit therefore combined cereal-based energy sources with three main sources of protein: legumes, canned fish, and powdered milk. A detailed nutritional analysis was not available to the research team, as procurement records were managed by the *Junta de Beneficencia* (Charity Board). Because receipt of the food kit was universal and did not vary across participants, it could not be entered as a covariate in the within-sample analysis and was therefore considered narratively as a co-intervention that the present design cannot disentangle from the effect of the home visits themselves.

The support process was guided by the principles of Family-Centered Practices (FCP), including collaborative partnerships with families, respect for family priorities, intervention within everyday routines, and the joint development of strategies adapted to each household’s circumstances (24, 28, 30–32). The intervention was delivered by five community health workers with prior technical training in nursing, early childhood education, or primary education, all of whom had previous experience in community-based work. They received specific training and ongoing supervision from the project’s technical team throughout the intervention period.

Training was provided in-service during the first 6 months of the program through biweekly 4-h sessions, totaling 48 h per community health worker. The training combined theoretical content on FCP with guided practice and direct support during home visits. Subsequently, ongoing reflective supervision was provided through biweekly 2-h sessions, which included a joint visit to a family followed by group case discussion (33). Each community health worker was assigned 42 families, consistent with the caseload established in comparable national programs implemented by the Ministry of Education and MIES. Home visits were initially scheduled on a biweekly basis over a six-month period (approximately 12 planned visits per family). The effective implementation period was 5 months, during which approximately 2,446 visits were completed, corresponding to an average of 2.35 visits per family per month (approximately 11.75 visits per family over the intervention period). Fewer than 2% of scheduled visits were canceled, primarily because of public holidays or temporary family circumstances that required families to be away from the community, indicating a very high level of adherence to the intervention.

No structured, independent fidelity-monitoring instrument (e.g., an observation checklist against core FCP components) was used during implementation; this is acknowledged as a limitation, and we recommend that future implementations incorporate one from the outset.

During the initial phase, families participated in a structured self-assessment process. This included the development of an ecogram to identify family and community support networks, as well as an exploration of their needs, concerns, and priorities regarding childcare and the prevention of chronic child malnutrition. Based on this process, each family identified priority areas for improvement, which were translated into functional goals related to feeding routines, hygiene practices, healthcare utilization, and access to community services.

Subsequent visits focused on reviewing progress towards these goals, promoting reflection on everyday practices, identifying barriers and facilitators to change, and collaboratively adjusting strategies according to each family’s circumstances and priorities.

#### Instruments and information sources

##### Knowledge, Practices, and Competencies Scale

The Knowledge, Practices, and Competencies Scale regarding the determinants of CCM was used (EPC-DCI; AUTHORS), designed *ad hoc* by the advisory team at Universidad Casa Grande. The instrument was reviewed by a nutrition specialist and a specialist in quantitative instrument development and was piloted with 70 mothers from a neighboring, non-participating community beforehand. No formal cognitive-interview protocol or content-validity index was used. Their feedback helped improve the clarity and comprehensibility of the scale, identify redundant items, and refine the items to optimize their contribution to the underlying construct. The improved scale consists of 34 items using a six-point Likert scale: (1 = Not at all true to 6 = completely true). It is organized into three dimensions: Knowledge (10 items; maximum score = 60), Practices (12 items; maximum = 72), and Competencies (12 items; maximum = 72). Because the instrument was developed specifically for this study, its results should be interpreted as contextual and exploratory indicators; additional psychometric analyses are required to more robustly establish its measurement properties. Internal consistency in the study sample was α = 0.783 (ω = 0.833) in the pre-intervention measurement and α = 0.814 (ω = 0.893) in the post-intervention measurement. These data constitute an initial psychometric assessment of the instrument, pending more extensive psychometric analyses. These data constitute an initial psychometric assessment of the instrument, pending more extensive psychometric analyses; domain-specific internal consistency and further detail on the item-to-domain structure are reported in Table 2 and in the Supplementary Scoring Appendix. The instrument was originally developed and administered in Spanish; the English item wording reported here (including in Table 2) reflects a translation prepared for manuscript purposes. The three domains comprise, respectively: Knowledge, items 6, 13, 15, 23, 25, 28, 29, 32–34 (10 items); Practices, items 1, 2, 5, 7, 8, 10, 11, 12, 14, 16, 20, 24 (12 items); and Competencies, items 3, 4, 9, 17–19, 21, 22, 26, 27, 30, 31 (12 items). No items are reverse-scored; domain and total scores are computed as the sum of available (non-missing) items. Item-level missingness varied substantially by item and time point and was especially high, at post-test, for items 12, 28, and 29 (128, 111, and 137 of 208 cases missing, respectively); this is discussed further in the Limitations.

**Table 2:** Pre- and post-test changes in the overall score and scores by dimension on the scale.

Variable	Pre FCP M (SD)	Post FCP M (SD)	M Diff.	95% CI	*t* (207)	*p*	dz
Total (34 items)	148.70 (18.95)	156.73 (17.89)	8.03	[4.53; 11.53]	−4.53	<0.001	0.31
Knowledge (10 items)	38.08 (8.45)	44.22 (5.59)	6.14	[4.86; 7.42]	−9.49	<0.001	0.66
Practices (12 items)	54.31 (7.06)	54.89 (7.57)	0.58	[−0.85; 2.01]	−0.80	0.425	0.06
Competences (12 items)	56.30 (7.54)	57.62 (7.66)	1.31	[−0.16; 2.80]	−1.76	0.080	0.12

*N* = 208. dz = Cohen’s d for paired samples. All values recomputed directly from the item-level dataset (paired *t*-tests on all pairwise-available data); Knowledge uses the corrected domain composition (see Methods). *t* and *p* correspond to a Post-minus-Pre difference throughout (positive = increase).

### Open-ended questions

A brief questionnaire consisting of open-ended questions was used as a qualitative supplement. This questionnaire was completed in writing by a subgroup of 25 randomly selected mothers. The questions explored perceived learnings, changes attributed to the support and the role of the health promoters. This source facilitated the compilation of meanings and illustrations that complemented the interpretation of the quantitative results that gives a voice to families, which is important in research into family-centered practices (34).

### Procedure

The initial measurement of the scale was conducted during the second home visit, corresponding to the operational start of the support process, and was designated Pre-FCP. The final measurement was administered at the end of the six-month intervention, during the last visit of the semester, and was designated Post-FCP. The promoters read the questions and answer options to the mothers and recorded the responses using Google Forms when internet access was available and using KOBO in households without connectivity. The open-ended questionnaire was administered in December, during the final food kit delivery. The information was provided on paper, written responses were requested, and the family identification code was used as the sole means of identification. Because the Pre-FCP measurement followed an initial contact visit devoted to rapport-building and a family self-assessment exercise, some early orientation to program content may already have occurred by the time of this “baseline” measurement; if anything, this would work against detecting a pre-post difference rather than inflating it and is noted as a limitation of the pre-post design rather than assumed to bias results in either direction. A subgroup of 25 mothers was selected from the larger pool of families enrolled in the program using predefined eligibility criteria. Mothers were eligible if they had participated in the program from its beginning, and selection sought to ensure variation in the ages of the participating children. Five mothers were selected from the caseload of each promoter, resulting in a total of 25 participants. Thus, the qualitative subsample was purposively selected to represent families with different child age profiles rather than being drawn through simple random sampling. All 25 selected mothers completed the questionnaire.

### Data analysis

Means (M) and standard deviations (SD) were calculated for the overall scale score and its dimensions at the Pre-FCP and Post-FCP time points. Paired *t*-tests were performed to examine pre-post changes. Normality of the paired differences was assessed with the Shapiro–Wilk test (satisfied for the Knowledge domain, *p* = 0.34; modestly violated, with skew near zero and some excess kurtosis, for the Total, Practices, and Competencies differences, *p* < 0.05), and Wilcoxon signed-rank tests are reported as a robustness check alongside the primary paired *t*-tests. Cohen’s dz. for paired samples (mean difference divided by the SD of the difference scores), with 95% confidence intervals, and the 95% confidence interval for the difference in means, were estimated. Given that 34 item-level tests were conducted, Holm-Bonferroni correction was applied across items; item-level findings are reported as exploratory. Analyses were also conducted at the nutritional status level (no anemia/CCM, anemia, anemia + CCM, CCM only) using the total scale score; a group-by-time interaction was estimated with a mixed-design ANOVA to formally test whether the magnitude of change differed across subgroups, and Holm-Bonferroni correction was applied across the four within-group comparisons. Because the community health worker who delivered the intervention also administered the outcome measures, and the analysis file does not retain a worker identifier, potential clustering by worker could not be evaluated; this is acknowledged as a limitation. A covariate-adjusted regression of the change score on baseline score, maternal age, pregnancy status, and child age was also fitted to characterize regression to the mean and rule out simple demographic confounding. Additionally, the percentage of participants who experienced improvements in the overall score was described. Open-ended responses were analyzed using a deductive approach, with the analysis organized around the categories of program appreciation, knowledge, practices and perceived competencies. Coding was performed by the project’s technical coordinator, reviewed by the project director, and cross-checked by an external researcher to reduce the risk of bias. Coding followed an *a priori* codebook built around four categories (program appreciation, knowledge, practices, and perceived competencies), with disagreements resolved by discussion; because initial coding was performed by program-affiliated staff, we acknowledge a residual risk of confirmation bias despite the independent review step. Data analysis coding is provided as Supplementary material.

## Results

Results are presented in the following order: participant flow and baseline characteristics; total and domain-level scores; item-level analysis by domain; subgroup analysis by nutritional status; and qualitative findings.

Internal consistency of the total scale was α = 0.783 (ω = 0.833) at Pre-FCP and α = 0.814 (ω = 0.893) at Post-FCP (see Methods—Instruments; domain-level reliability is reported in the Supplementary Scoring Appendix). These values are reported here as a data-quality descriptive only, without interpreting the modest pre-post change as a substantive finding.

The total score on the scale increased from 148.70 (SD = 18.95) at baseline to 156.73 (SD = 17.89) post-intervention. The mean difference was 8.03 points (95% CI [4.53; 11.53]), which was statistically significant (*t*(207) = −4.53, *p* < 0.001), with a small effect size (dz = 0.31; see Table 2). These results show that, when looking at the whole scale, the support was linked to a small but important increase (see Table 1).

The analysis by dimension reveals a differentiated pattern, with the most robust increase occurring in the Knowledge dimension (d = 0.66), while Practices (d = 0.06, *p* = 0.425, 0.8% change) and Competencies (d = 0.12, dz. = 0.12, *p* = 0.080, 1.8% change) showed more modest and non-significant increases in the aggregate analysis.

### Item-by-item analysis

This analysis allows us to identify more precisely which specific program content produced the most consistent changes.

Table 3 shows the changes in scores for the 34 items on the scale before and after the program. In total, 22 of the 34 items showed statistically significant changes (*p* < 0.05). At the uncorrected α = 0.05 level, 22 of the 34 items showed statistically significant changes; 16 of these 34 remained significant after Holm-Bonferroni correction for multiple comparisons, and item-level findings are reported as exploratory. The largest effect sizes were concentrated in items 32, 33, and 34 (dz-values of 0.891, 0.859, and 0.903, respectively), corresponding to knowledge of the consequences of CCM, preventive strategies, and coping with the diagnosis. The next-largest effect sizes were found in items 28 and 29 (dz = 0.76 and 0.83) both based on substantially smaller valid samples (*n* = 84 and 61 of 208, respectively, owing to high post-test missingness) and item 12 (dz = 0.72, *n* = 71). Two items showed statistically significant score decreases at the uncorrected level: item 6 (“I need more knowledge to improve my family’s nutrition,” *p* = 0.019) and item 26 (“I make sure my child gets all recommended vaccinations,” *p* = 0.014); neither remained significant after Holm-Bonferroni correction.

**Table 3:** Pre-post statistics for the 34 items of the scale.

#	Item (*n* = valid pairs)	*n*	Pre M (SD)	Post M (SD)	M diff. [95% CI]	*t* (df)	*p*	*p* (Holm)	dz
Knowledge domain (10 items)									
6	I need more knowledge to improve my family’s nutrition.	207	5.30 (0.65)	5.12 (0.80)	−0.18 [−0.33, −0.03]	−2.36 (206)	0.019	0.287	−0.16
13	I received adequate information about breastfeeding.	188	4.40 (1.49)	5.03 (0.90)	0.63 [0.39, 0.87]	5.09 (187)	<0.001	<0.001	0.37
15	I know the benefits of continuing breastfeeding for 2 years.	203	4.23 (1.58)	5.12 (0.85)	0.90 [0.67, 1.13]	7.67 (202)	<0.001	<0.001	0.54
23	I have the knowledge to take good care of my children.	205	4.91 (0.86)	5.13 (0.77)	0.22 [0.07, 0.38]	2.81 (204)	0.006	0.094	0.20
25	I believe I need to learn more about children’s overall development and health.	203	4.70 (1.14)	5.10 (0.86)	0.40 [0.21, 0.59]	4.20 (202)	<0.001	0.001	0.30
28	I know what to do if my child has experienced or experiences stunted growth. [wording corrected]	84	2.94 (2.09)	4.74 (1.10)	1.80 [1.28, 2.31]	6.93 (83)	<0.001	<0.001	0.76
29	I know what to do if my child has experienced or experiences low weight. [wording corrected]	61	2.54 (1.95)	4.51 (1.29)	1.97 [1.36, 2.57]	6.51 (60)	<0.001	<0.001	0.83
32	We know the problems our child can have in case of CCM.	206	3.52 (1.66)	5.12 (0.73)	1.61 [1.36, 1.86]	12.78 (205)	<0.001	<0.001	0.89
33	We know how to prevent chronic malnutrition.	205	3.52 (1.64)	5.13 (0.83)	1.61 [1.35, 1.87]	12.30 (204)	<0.001	<0.001	0.86
34	I have the necessary information about what to do if our child experiences CCM.	205	3.44 (1.60)	5.10 (0.81)	1.65 [1.40, 1.91]	12.93 (204)	<0.001	<0.001	0.90
Practices domain (12 items)									
1	Our family has a stable and adequate food supply.	197	4.37 (0.92)	4.68 (0.88)	0.31 [0.13, 0.48]	3.38 (196)	0.001	0.017	0.24
2	We regularly share a daily meal together.	201	5.12 (0.80)	5.06 (0.85)	−0.07 [−0.24, 0.10]	−0.81 (200)	0.420	1.000	−0.06
5	My daily meals include a variety of foods.	194	4.27 (1.21)	4.84 (0.98)	0.57 [0.36, 0.78]	5.38 (193)	<0.001	<0.001	0.39
7	As a mother, I eat a healthy and adequate diet.	206	4.17 (1.23)	4.68 (1.08)	0.52 [0.30, 0.73]	4.68 (205)	<0.001	<0.001	0.33
8	As a mother, my daily diet includes a variety of foods.	204	4.18 (1.21)	4.81 (0.99)	0.63 [0.42, 0.84]	5.82 (203)	<0.001	<0.001	0.41
10	I pay attention to my children’s hygiene during meals.	203	5.43 (0.59)	5.32 (0.70)	−0.12 [−0.25, 0.01]	−1.80 (202)	0.074	0.883	−0.13
11	During my pregnancy, I had a nutritious and healthy diet.	206	4.60 (1.07)	4.76 (1.00)	0.16 [−0.05, 0.37]	1.53 (205)	0.129	1.000	0.11
12	I know the recommendations for introducing complementary foods safely and nutritiously.	71	3.14 (1.98)	4.66 (1.21)	1.52 [1.02, 2.02]	6.05 (70)	<0.001	<0.001	0.72
14	My child was breastfed for more than 6 months.	166	5.35 (0.94)	5.28 (0.82)	−0.07 [−0.26, 0.13]	−0.67 (165)	0.506	1.000	−0.05
16	Starting at 6 months, my child received complementary foods.	155	5.10 (1.12)	5.21 (0.77)	0.10 [−0.13, 0.33]	0.89 (154)	0.374	1.000	0.07
20	My child has regular access to medical checkups.	187	5.40 (0.66)	5.26 (0.77)	−0.14 [−0.29, 0.02]	−1.77 (186)	0.078	0.883	−0.13
24	I practice proper food hygiene to prevent illness.	208	5.35 (0.73)	5.37 (0.53)	0.01 [−0.12, 0.15]	0.21 (207)	0.833	1.000	0.02
Competencies domain (12 items)									
3	I am satisfied with the food I serve at the table.	206	4.55 (1.12)	5.04 (0.91)	0.49 [0.29, 0.69]	4.93 (205)	<0.001	<0.001	0.34
4	I feel that I feed my family properly.	208	4.36 (1.11)	4.88 (0.91)	0.52 [0.33, 0.71]	5.26 (207)	<0.001	<0.001	0.37
9	I know what to do when my child displays challenging behaviors during mealtimes.	181	4.56 (1.29)	4.84 (0.94)	0.28 [0.04, 0.51]	2.32 (180)	0.021	0.297	0.17
17	I have had a positive breastfeeding experience.	179	5.08 (1.08)	4.93 (0.98)	−0.15 [−0.39, 0.09]	−1.25 (178)	0.212	1.000	−0.09
18	I know how to provide healthy nutrition for my child.	205	4.97 (0.95)	5.11 (0.94)	0.15 [−0.04, 0.33]	1.54 (204)	0.126	1.000	0.11
19	I can proactively manage my own health.	195	4.95 (1.13)	5.23 (0.73)	0.28 [0.09, 0.47]	2.85 (194)	0.005	0.087	0.20
21	My family enjoys good health.	205	4.42 (1.18)	4.88 (0.93)	0.46 [0.26, 0.67]	4.46 (204)	<0.001	<0.001	0.31
22	My child is currently in good health.	190	4.80 (1.00)	4.96 (0.95)	0.17 [−0.04, 0.37]	1.62 (189)	0.107	0.964	0.12
26	I make sure my child gets all recommended vaccinations.	187	5.55 (0.50)	5.41 (0.62)	−0.14 [−0.26, −0.03]	−2.49 (186)	0.014	0.219	−0.18
27	As a mother, I make sure my child attends medical appointments for growth/weight monitoring.	184	5.52 (0.58)	5.41 (0.60)	−0.11 [−0.23, 0.02]	−1.74 (183)	0.084	0.883	−0.13
30	I did everything necessary to stay healthy during my pregnancy.	192	4.35 (1.49)	4.62 (1.04)	0.27 [0.00, 0.54]	2.00 (191)	0.047	0.609	0.14
31	Our family receives support from organizations promoting child well-being.	192	5.02 (1.14)	4.98 (0.90)	−0.04 [−0.24, 0.16]	−0.41 (191)	0.683	1.000	−0.03

*** *p* < 0.001, ** < 0.01; * < 0.05; Positive *t* values indicate a post-decrease. No items are reverse-scored. Items 28/29 wording corrected (see Methods); both show high post-test missingness (111/137 of 208 cases), hence the much smaller valid *n*. *p*(Holm) applies Holm-Bonferroni correction across all 34 items (16 of 34 remain significant after correction). *t* and mean differences are Post-minus-Pre throughout (positive = increase).

### Subgroup analysis by nutritional status

The analysis of pre-post changes based on the nutritional status reported in the household allows us to examine whether the effect of the support program differed among families depending on their baseline situation. The results are presented in Table 4. The subgroup sizes were: no anemia/CCM, *n* = 116; anemia only, *n* = 25; CCM only, *n* = 26; anemia + CCM, *n* = 40 (*n* = 207; 1 missing). Within-group changes were statistically significant for the no-anemia/CCM group (dz = 0.31), the CCM-only group (dz = 0.43), and the anemia + CCM group (dz = 0.55), but not for the anemia-only group (dz = −0.07, *p* = 0.731). However, a formal group-by-time interaction test on the total score was not statistically significant, *F*(3, 204) = 2.09, *p* = 0.100, so these subgroup differences should be read as descriptive and hypothesis-generating rather than as evidence that the intervention was differentially effective.

**Table 4:** Pre-post change in the total scale score by nutritional-status subgroup.

Subgroup	*n*	Pre-FCP M (SD)	Post-FCP M (SD)	Mean diff. [95% CI]	*t* (df)	*p*	*p* (Holm)	dz
No anemia or CCM	116	148.32 (18.68)	156.16 (18.87)	7.84 [3.19, 12.48]	3.34 (115)	0.001	0.004	0.31
Anemia only	25	153.72 (22.58)	151.72 (17.53)	−2.00 [−13.88, 9.88]	−0.35 (24)	0.731	0.731	−0.07
CCM only	26	150.19 (13.69)	158.96 (16.61)	8.77 [0.54, 17.00]	2.19 (25)	0.038	0.076	0.43
Anemia + CCM	40	145.65 (20.36)	160.28 (15.74)	14.62 [6.10, 23.15]	3.47 (39)	0.001	0.004	0.55

*N* = 207 with valid subgroup classification (1 missing). Reconciled directly from the Anemia DCI variable; the previously submitted table mislabeled subgroups and omitted the anemia + CCM group (the largest, *n* = 40) entirely. Group-by-time interaction (mixed-design ANOVA on total score): *F*(3, 204) = 2.09, *p* = 0.100—not significant; subgroup differences are descriptive/exploratory, not evidence of differential effectiveness.

### Knowledge dimension

Changes to the knowledge dimension were the most consistent. As shown in Table 4, the items with the greatest increase were the benefits of extending breastfeeding until the age of two (I15, d = 0.54); knowledge of the problems that CCM can cause (I32, d = 0.89); knowledge of prevention strategies (I33, d = 0.86); and information on how to respond to a possible CCM diagnosis (I34, d = 0.90).

The open-ended responses provide further insight into this finding. Several mothers explicitly mentioned that they had incorporated information about safe water as a preventive measure against CCM, saying “Thanks to the visits, I know how important safe water is”; “Everyone in my family drinks safe water”; and “safe water and healthy eating help prevent CCM.” Furthermore, references were made to enhanced clarity regarding the response to indications of CCM or anemia, with statements denoting both declarative knowledge and a propensity to act. These item-level results are exploratory and should be interpreted alongside the multiple-comparisons correction reported above.

### Practices dimension

Although the Practices dimension did not show a statistically significant overall change, item-by-item analysis reveals significant variations in three specific behaviors related to dietary diversity, with scores showing improvements with mall effect sizes (I5, d = 0.39); self-assessment of eating adequately (I7, d = 0.33); and the perception of consuming a varied diet (I8, d = 0.41). Item I12 (family dietary variety) also showed a medium-to-large effect size (d = 0.72, *n* = 71); items 28 and 29 (knowledge domain, not Practices—see corrected item-to-domain mapping in Methods) showed the largest effect sizes in the full item set (d = 0.76 and 0.83) but are based on much smaller valid samples (*n* = 84 and 61) owing to high post-test missingness, and their wording has been corrected (see Table 3 footnote).

The open-ended responses supplement these results with specific examples of changes initiated by the mothers themselves, which provide further insight into the initiatives that have been implemented. Some participants described how they had incorporated legumes into their daily diet: “More nutritious foods every day: lentils and chickpeas”; “With beans, I have a more varied diet.” Other families mentioned changes to hygiene practices relating to the use of clean water when preparing food. These descriptions are consistent with the type of changes facilitated by the home visit model: gradual adjustments to existing routines based on reflection and support rather than radical habit change.

### Competence dimension

At the uncorrected level, seven of the twelve Competencies items showed statistically significant change: satisfaction with food served to family members (I3, d = 0.34); perception of properly feeding family members (I4, d = 0.37); application of knowledge of healthy eating in early childhood (I18, d = 0.11); knowing what to do when a child displays challenging mealtime behaviors (I9, d = 0.17); proactive management of one’s own health (I19, d = 0.20); perception of family health (I21, d = 0.31); staying healthy during pregnancy (I30, d = 0.14); and a statistically significant decrease in ensuring recommended vaccinations (I26, d = −0.18). After Holm-Bonferroni correction, only three of these remain significant: I3, I4, and I21. Item 18 (application of knowledge of healthy eating, d = 0.11) was not statistically significant (*p* = 0.126) and is not counted among the significant items. There was also a numerical decrease in the item regarding the positive experience of breastfeeding (I17, from 5.08 to 4.94), which was not statistically significant (*p* = 0.212) and is presented here only as a descriptive observation, not as a finding. The open-ended responses showed that some mothers attributed greater confidence in selecting, preparing, and serving food to the process: “Now I serve a balanced and proper diet”; “I prepare new recipes”; and “I’ve incorporated foods that my child did not eat before.” These statements reflect a subjective sense of ownership of the process and suggest signs of strengthened parental self-efficacy—an outcome that the FCP identifies as a key mediator of changes in practices (17, 27) although they do not replace direct observation of behaviors.

## Discussion

The present study described the pre-post changes observed after 6 months of family support guided by a Family-Centered Practices (FCP) approach adapted to a rural context of high social vulnerability in Ecuador. The total and Knowledge scale scores increased significantly, while the aggregate Practices (*p* = 0.425) and Competencies (*p* = 0.080) scores did not change significantly, although statistically significant improvements were recorded in specific items within each domain. The main finding was the significant increase in the Knowledge dimension (dz = 0.66), in the moderate effect-size range. This differentiated pattern is relevant because evidence from family- and caregiver-oriented interventions indicates that knowledge, perceived competence, and everyday practices represent related but distinct outcomes that may respond differently to intervention and contextual conditions (17, 19, 20).

### Knowledge gains

Interventions aimed at modifying complex family behaviors may initially produce more readily detectable changes in knowledge and awareness than in established daily practices. Previous nutrition and family-engagement research has shown improvements in caregiver knowledge following educational and behavior-change interventions, while changes in practices tend to be more variable and dependent on contextual conditions (18–20). Particularly relevant to the present findings, Setiawan et al. (35) reported improvements in maternal knowledge, attitudes, and self-efficacy following a family-based intervention focused on stunting prevention. Although the intervention, design, and sociocultural context differ from those of the present study, the convergence in knowledge-related outcomes supports the relevance of actively involving caregivers in the prevention of child malnutrition. From this perspective, the strengthening of knowledge observed in the present study may constitute an important early outcome, although the current design does not establish that such knowledge will subsequently translate into sustained changes in practices.

Subgroup analysis by nutritional status showed that within-group change was statistically significant for three of the four groups (no anemia/CCM, CCM only, and anemia + CCM) but not for the anemia-only group. However, a formal group-by-time interaction test was not statistically significant, *F*(3, 204) = 2.09, *p* = 0.100, so these patterns should not be interpreted as evidence of a differentially larger or “especially effective” response in any subgroup. A covariate-adjusted analysis showed a strong negative association between baseline score and the amount of change (b = −0.95, *p* < 0.001), consistent with substantial regression to the mean, which offers a more parsimonious explanation for the pattern across subgroups than a differential-effectiveness account. These subgroup findings should therefore be considered descriptive and hypothesis-generating and require replication with larger, adequately powered samples.

### Item-level patterns

Item-level analysis provides a more nuanced perspective than aggregate analysis by dimension. Twenty-two of the 34 items showed statistically significant changes at the uncorrected level, and 16 remained significant after Holm-Bonferroni correction. This broader-than-dimensional pattern should be interpreted cautiously given the number of comparisons and the measurement limitations discussed above. The highest effect sizes were concentrated in items related to specific knowledge about CCM and in selected eating-related behaviors. This pattern is compatible with the logic of family-centered support, in which intervention strategies are developed around specific family priorities, goals, and everyday routines rather than necessarily producing uniform changes across all areas (17, 26, 31).

The reduction in knowledge-score dispersion adds an important nuance to the increase in mean scores. Descriptively, participants with lower initial knowledge scores tended to show larger pre-post changes, resulting in less variability at post-test. However, this finding should be interpreted cautiously in light of the strong relationship between baseline scores and change identified in the regression-to-the-mean analysis. Therefore, the narrowing of score dispersion cannot be attributed specifically to the support process. Nevertheless, the pattern highlights the importance of considering participants’ starting points when evaluating change in community-based interventions.

### Practices and competencies

The statistically significant changes observed in specific items within the Practices and Competencies domains are consistent with, although they do not by themselves establish, a possible role of the support process in fostering reflection and initial adjustments in everyday feeding and caregiving routines. Given the uncontrolled design, this represents one plausible interpretation rather than a demonstrated intervention effect.

The absence of statistically significant change in the aggregate Practices score is consistent with evidence indicating that nutritional behavior change is often uneven across particular practices and that its sustainability depends on factors extending beyond knowledge and individual family capacities. Household resources, food security, access to nutritious foods, income, social support, and opportunities to implement recommended behaviors can all facilitate or constrain change (8, 9, 20, 36). This is particularly relevant in the Ecuadorian context, where chronic child malnutrition has been described as a multifactorial phenomenon associated with interacting socioeconomic, environmental, maternal, care-related, and health-service conditions (11). Evidence from Ecuador has also highlighted relationships between social and community resources and children’s nutritional status (13), as well as associations between feeding practices and stunting in vulnerable populations (14).

From this perspective, the findings suggest that translating knowledge and guidance into sustained behavioral change requires not only educational support and follow-up but also material and social conditions that enable families to maintain recommended practices over time. The absence of statistically significant aggregate change in Practices or Competencies should therefore not be interpreted as equivalent to the absence of any family-level process, although neither can such processes be inferred solely from significant changes in individual items.

This differentiated pattern is also consistent with the conceptual logic of FCP. Family-centered interventions build on each family’s routines, strengths, priorities, and circumstances, and therefore may produce heterogeneous outcomes depending on families’ starting points and contextual conditions (26, 31). Contemporary evidence further emphasizes the combination of relational and participatory practices as a defining feature of family-centered support and links these practices with family empowerment, parenting capacity, achievement of family goals, and satisfaction with services (17). Research in early intervention has also identified family confidence as a relevant family outcome associated with broader family quality-of-life processes (27). Moreover, experience with FCP in rural and socially vulnerable Ecuadorian contexts supports the feasibility of adapting professional support to families’ priorities and natural environments (16).

Accordingly, the absence of significant change in some areas may reflect the greater complexity involved in modifying established behaviors, as well as the need for time, structural support, or material resources. However, because the present study did not include a comparison group or direct observational measures of family practices, it cannot determine whether the item-level changes were attributable to FCP.

The numerical decline in the item concerning positive breastfeeding experiences also warrants consideration, but it should not be overinterpreted because the change was not statistically significant. One possible explanation is that increased knowledge may influence how participants retrospectively evaluate previous feeding experiences; however, the present data do not allow this interpretation to be tested.

### Qualitative insights

The open-ended qualitative responses provide an additional layer of meaning that the quantitative data do not fully capture. Repeated references to safe water as a preventive factor, mentions of specific changes in the incorporation of legumes and vegetables, and expressions of greater confidence in feeding the family suggest that some participants perceived changes that were meaningful within their everyday routines.

These narratives are consistent with outcomes that FCP seek to promote: not merely the acquisition of information, but also active family involvement and increased confidence in responding to everyday situations (17, 23, 27). They are also consistent with recent research emphasizing the importance of including families’ own perspectives when examining Family-Centered Practices and support in natural environments (34). However, because these accounts were obtained from a subgroup of 25 participants through brief written responses, their interpretive scope is necessarily limited. They should therefore be considered complementary to the quantitative findings rather than independent evidence of behavioral change.

### Interpretation and alternative explanations

Interpreting the overall results requires acknowledging a key limitation: family support was implemented as part of a program that also included the monthly distribution of a food kit. This combination of components, which is characteristic of many community-based responses in vulnerable contexts, makes it impossible to attribute the observed changes exclusively to the support process under the FCP approach.

One possible, untested explanation is that the observed changes reflect an interaction between the relational and reflective support provided through home visits and the material support provided by the food kits. The present design cannot establish or rule out such a synergy, and this interpretation therefore remains speculative. Nevertheless, combining support for caregiving capacities with interventions addressing material and nutritional conditions is consistent with contemporary approaches emphasizing the multidimensional nature of child malnutrition (4, 8–10). The home-visiting format also provides a context in which support can be linked to families’ actual routines and living conditions; evidence from the large-scale Cuna Más program in rural Peru indicates that structured home visiting can produce modest improvements in child development and selected parenting behaviors when implemented through community services (21). However, the objectives and intervention content of that program differ from those of the present study.

Other alternative explanations for the observed pre-post changes also deserve consideration and cannot be ruled out by this design, including regression to the mean, for which direct evidence was found in the present analyses, repeated-test familiarity, maturation, changing child age, external healthcare exposure, seasonal food availability, social-desirability bias, interviewer expectancy because outcome data were collected by the same workers who delivered the intervention, and Hawthorne effects.

A statistically significant change in a non-validated, study-specific self-report score does not necessarily represent a clinically or programmatically meaningful improvement in child nutrition or family functioning. In the absence of a validated instrument, an external minimal-important-difference benchmark, or objective child outcomes, this study can describe statistical change but cannot establish clinical or public-health significance. No anthropometric, biochemical, dietary-intake, or child-development data were collected, so the study cannot determine whether changes in caregiver-reported knowledge were accompanied by objective improvement in child status. Accordingly, the implications discussed below are limited to feasibility and hypothesis generation and do not extend to recommendations for policy or scale-up.

### Implications

The present study contributes to an emerging area of research by exploring the application of FCP in a community-based program aimed at preventing and addressing chronic child malnutrition. In Ecuador, chronic child malnutrition and anemia have been described as multifactorial public health problems associated with socioeconomic, environmental, biological, care-related, community, and health-service factors (11, 13, 14). These findings reinforce the importance of situating family caregiving practices within the broader material and social conditions in which families live rather than interpreting them as isolated individual behaviors.

Most evidence supporting FCP originates from early intervention services for children with disabilities or developmental-risk conditions (22, 25, 28), where the approach is well established. More recent evidence has strengthened this empirical basis by identifying relational and participatory practices as key components of family-centered intervention and linking them to a range of family outcomes (17). Research on the implementation of recommended practices has also emphasized the importance of structured implementation processes, professional training, collaboration, and ongoing support when translating family-centered principles into routine service delivery (29). Evidence from Ecuador has documented the implementation of FCP with children and families living in rural and socially vulnerable contexts (16).

Extending this approach specifically to nutrition and community health therefore represents a theoretically supported but still insufficiently tested application. The present findings are relevant to both community health and family-intervention research because they provide preliminary evidence regarding the feasibility of incorporating family-centered support into a broader program addressing CCM and identify caregiver-related outcomes that may be sensitive to change. At the same time, they do not establish that FCP are effective in preventing or reducing CCM.

Future studies should use more robust research designs capable of establishing causal relationships and examining longer-term outcomes. Controlled or pragmatic comparative designs, validated measures of caregiver knowledge, practices and perceived competence, independent outcome assessment, systematic evaluation of implementation fidelity, and objective nutritional and developmental indicators would allow the contribution of family-centered support to be examined more clearly. Future research could also explore how structural, community, and family-level conditions influence families’ ability to translate knowledge into sustained practices.

## Conclusion

After 6 months of participation in an integrated home-visiting and food-support program, mothers reported higher knowledge scores related to the prevention and management of chronic child malnutrition, with a moderate effect size (dz = 0.66) and reduced variation among participants—the most consistent and statistically robust result of this study.

Aggregate Practices (*p* = 0.425) and Competencies (*p* = 0.080) scores did not change significantly, although some individual items within these domains showed statistically significant change at the uncorrected level. This does not necessarily imply the complete absence of change in these areas, but it does mean that, unlike the Knowledge dimension, an aggregate effect on Practices and Competencies is not supported by this study.

Because the study lacked a comparison group and used a non-validated, study-specific self-report instrument, these findings should be considered preliminary and hypothesis-generating. Controlled evaluations with validated outcome measures, independent outcome assessment, and objective child-nutrition indicators are needed before conclusions about the effectiveness of the Family-Centered Practices approach for chronic child malnutrition prevention can be drawn.

### Limitations

The results should be interpreted in light of several limitations. First, the pre-post design without a comparison group prevents us from establishing causal relationships or ruling out alternative explanations for the observed changes, such as natural maturation, secular trends, seasonal variation, or the effect of other program components, including the concurrent monthly food kits, from which the effect of the FCP home-visiting component cannot be separated. Second, the qualitative component relied on brief written responses from 25 participants, which considerably limits the depth of interpretation compared to what an in-depth interview or focus-group design would allow, and may have disadvantaged participants with limited literacy. Third, nutritional status was based on caregiver report rather than anthropometric or biochemical measurement, and should be treated as indicative rather than diagnostic. Fourth, outcome data were collected by the same community workers who delivered the intervention, raising the risk of social-desirability and expectancy effects that would tend to inflate the observed improvement; future evaluations should use independent, blinded outcome assessment. Fifth, the instrument used is study-specific and its structural and construct validity have not been established; all findings based on it, including the total score, should be read as exploratory. Sixth, 34 item-level and four subgroup comparisons were tested; we applied Holm-Bonferroni correction, but residual risk of false-positive findings remains, and item-level results should not be read as validated changes in family capacities. Seventh, item-level missingness was substantial and uneven across items and time points (notably for items 12, 28, and 29), and reliability/effect-size estimates for the total scale and for these items rest on much smaller effective samples than the nominal *N* = 208. Eighth, potential clustering of outcomes by community health worker could not be evaluated because a worker identifier was not retained in the analysis file. Ninth, the exclusion of 50 later-enrolling mothers, though decided prospectively, could not be checked against the analyzed sample for baseline comparability and may limit representativeness. Tenth, no intervention-fidelity data were collected, so consistency of delivery across families and workers cannot be verified.

Additionally, the researchers did not include objective measures of nutritional change, such as anthropometric indicators for children, or child development indicators. This limits the ability to assess the program’s ultimate impact on the outcomes that ultimately matter.

Subgroup analysis by nutritional status should be interpreted with caution given the size of some subgroups. These results are exploratory in nature and generate hypotheses that must be tested in studies with greater statistical power. Future studies could analyze causal attribution of the effect of support versus the dietary component. Furthermore, we encourage conducting a psychometric evaluation of the instrument, including convergent validity with already validated scales of parenting competencies and nutritional knowledge, as well as measurement invariance across groups. Finally, we aim to replicate this study in other rural Latin American contexts with different sociodemographic characteristics to assess the model’s generalizability. Finally, no anthropometric, biochemical, dietary-intake, or child-development indicators were collected, so this study cannot determine whether the observed changes in caregiver-reported knowledge were accompanied by any objective improvement in child nutritional status.

## Data Availability

The raw data supporting the conclusions of this article will be made available by the authors, without undue reservation.
