# Supplementary material for: Changes in family knowledge, practices, and perceived competencies after a family-centered home-visiting program for chronic child malnutrition prevention in rural Ecuador: an uncontrolled pre-post study
Source: Front Public Health. 2026 Oct 26;14:1925365. doi: 10.3389/fpubh.2026.1925365 (PMC13745657; doi:10.3389/fpubh.2026.1925365)
Supplement: Supplementary file 1 [file Data_Sheet_1.PDF]

```r
#!/usr/bin/env Rscript
#
===========================================================================
# Statistical analysis: Changes in family knowledge, practices, and
# perceived competencies after a family-centered home-visiting program
# for chronic child malnutrition prevention in rural Ecuador
#
# Data: EPC-DCI_anonymised_item_data.csv (208 participants, 34 items × 2 time
#      points, plus five demographic variables)
# Output: CSV files written to ./output/ and a consolidated Excel workbook
#
# Software requirements:
#   R >= 4.1.0
#   Packages: psych, openxlsx, afex, car
#
#   install.packages(c("psych", "openxlsx", "afex", "car"))
#
# Run from the project root:
#   Rscript EPC-DCI_analysis.R
#
===========================================================================

suppressMessages({
  library(psych)
  library(openxlsx)
  library(afex)
  library(car)
```

```r
})

options(warn = -1)

# ------------------------------------------------------------------------
# File paths
# ------------------------------------------------------------------------
script_dir <- tryCatch({
  dirname(sub("--file=", "", grep("--file=", commandArgs(FALSE), value = TRUE)))
}, error = function(e) getwd())
if (!nchar(script_dir)) script_dir <- getwd()

data_file <- file.path(script_dir, "EPC-DCI_anonymised_item_data.csv")
out_dir   <- file.path(script_dir, "output")
dir.create(out_dir, showWarnings = FALSE, recursive = TRUE)

# ------------------------------------------------------------------------
# 1. Load data
# ------------------------------------------------------------------------
dat <- read.csv(data_file, stringsAsFactors = FALSE)
cat(sprintf("Data loaded: %d participants, %d variables\n", nrow(dat), ncol(dat)))

# ------------------------------------------------------------------------
# 2. Item-to-domain mapping
#
# The EPC-DCI comprises 34 items organised into three content domains.
# Item numbers follow the original instrument numbering.
#
```

```
# NOTE: The SPSS output file shipped with the raw data contained an error
# in the Knowledge domain composition (it used the original 8-item version
# rather than the corrected 10-item version). Domain scores are therefore
# recomputed here directly from the item columns.
# ------------------------------------------------------------------------
DOMAINS <- list(
  Knowledge    = c(6, 13, 15, 23, 25, 28, 29, 32, 33, 34),   # 10 items
  Practices    = c(1, 2, 5, 7, 8, 10, 11, 12, 14, 16, 20, 24), # 12 items
  Competencies = c(3, 4, 9, 17, 18, 19, 21, 22, 26, 27, 30, 31) # 12 items
)
ALL_ITEMS <- 1:34

pre_col  <- function(i) sprintf("P%dPREPCF",  i)
post_col <- function(i) sprintf("P%dPOSTPCF", i)

# Item wording for labelling output tables
ITEM_LABELS <- c(
  "1"  = "Our family has a stable and adequate food supply.",
  "2"  = "We regularly share a daily meal together.",
  "3"  = "I am satisfied with the food I serve at the table.",
  "4"  = "I feel that I feed my family properly.",
  "5"  = "My daily meals include a variety of foods.",
  "6"  = "I need more knowledge to improve my family's nutrition.",
  "7"  = "As a mother, I eat a healthy and adequate diet.",
  "8"  = "As a mother, my daily diet includes a variety of foods.",
  "9"  = "I know what to do when my child displays challenging behaviors during
mealtimes.",
  "10" = "I pay attention to my children's hygiene during meals.",
```

"11" = "During my pregnancy, I had a nutritious and healthy diet.",

"12" = "I know the recommendations for introducing complementary foods safely and nutritiously.",

"13" = "I received adequate information about breastfeeding.",

"14" = "My child was breastfed for more than six months.",

"15" = "I know the benefits of continuing breastfeeding for two years.",

"16" = "Starting at 6 months, my child received complementary foods.",

"17" = "I have had a positive breastfeeding experience.",

"18" = "I know how to provide healthy nutrition for my child.",

"19" = "I can proactively manage my own health.",

"20" = "My child has regular access to medical checkups.",

"21" = "My family enjoys good health.",

"22" = "My child is currently in good health.",

"23" = "I have the knowledge to take good care of my children.",

"24" = "I practice proper food hygiene to prevent illness.",

"25" = "I believe I need to learn more about children's overall development and health.",

"26" = "I make sure my child gets all recommended vaccinations.",

"27" = "As a mother, I make sure my child attends medical appointments for growth/weight monitoring.",

"28" = "I know what to do if my child has experienced or experiences stunted growth.",

"29" = "I know what to do if my child has experienced or experiences low weight.",

"30" = "I did everything necessary to stay healthy during my pregnancy.",

"31" = "Our family receives support from organizations promoting child well-being.",

"32" = "We know the problems our child can have in case of CCM.",

"33" = "We know how to prevent chronic malnutrition.",

"34" = "I have the necessary information about what to do if our child experiences CCM."

```r
)

# -------------------------------------------------------------------------
# 3. Compute domain and total scores (sum of available, non-missing items)
# -------------------------------------------------------------------------
row_sum_avail <- function(mat) {
  apply(mat, 1, function(r) if (all(is.na(r))) NA_real_ else sum(r, na.rm = TRUE))
}

for (d in names(DOMAINS)) {
  dat[[paste0(d, "_PRE")]]  <- row_sum_avail(dat[, sapply(DOMAINS[[d]], pre_col),
drop = FALSE])
  dat[[paste0(d, "_POST")]] <- row_sum_avail(dat[, sapply(DOMAINS[[d]], post_col),
drop = FALSE])
}
dat$TOTAL_PRE  <- row_sum_avail(dat[, sapply(ALL_ITEMS, pre_col),  drop =
FALSE])
dat$TOTAL_POST <- row_sum_avail(dat[, sapply(ALL_ITEMS, post_col), drop =
FALSE])

write.csv(dat, file.path(out_dir, "00_dataset_with_computed_scores.csv"),
row.names = FALSE)
cat("Full dataset with computed domain/total scores saved to output/\n")

# -------------------------------------------------------------------------
# 4. Helper: paired-sample statistics (t-test, Cohen's dz, Shapiro-Wilk,
#    Wilcoxon signed-rank)
# -------------------------------------------------------------------------
paired_stats <- function(pre, post, label) {
  ok    <- complete.cases(pre, post)
```

```
  pre   <- pre[ok]
  post  <- post[ok]
  n     <- length(pre)
  d     <- post - pre
  md    <- mean(d)
  sdd   <- sd(d)
  tt    <- t.test(post, pre, paired = TRUE)
  dz    <- md / sdd

  sw <- if (n >= 3 & n <= 5000) {
    tryCatch(shapiro.test(d), error = function(e) list(statistic = NA, p.value = NA))
  } else {
    list(statistic = NA, p.value = NA)
  }
  wil <- tryCatch(wilcox.test(post, pre, paired = TRUE), error = function(e)
list(statistic = NA, p.value = NA))

  data.frame(
    scale      = label,
    n          = n,
    pre_M      = mean(pre),
    pre_SD     = sd(pre),
    post_M     = mean(post),
    post_SD    = sd(post),
    mean_diff  = md,
    ci95_low   = tt$conf.int[1],
    ci95_high  = tt$conf.int[2],
    t_stat     = unname(tt$statistic),
```

```r
    df        = unname(tt$parameter),
    p         = tt$p.value,
    dz        = dz,
    shapiro_W  = unname(sw$statistic),
    shapiro_p  = sw$p.value,
    wilcoxon_W = unname(wil$statistic),
    wilcoxon_p = wil$p.value
  )
}


# ------------------------------------------------------------------------
# 5. Total and domain scores: paired t-test, Cohen's dz
#    Reproduces Table 2 in the manuscript
# ------------------------------------------------------------------------
rows <- list(paired_stats(dat$TOTAL_PRE, dat$TOTAL_POST, "Total (34 items)"))
for (d in names(DOMAINS)) {
  rows[[length(rows) + 1]] <- paired_stats(
    dat[[paste0(d, "_PRE")]], dat[[paste0(d, "_POST")]],
    sprintf("%s (%d items)", d, length(DOMAINS[[d]]))
  )
}
scale_df <- do.call(rbind, rows)
write.csv(scale_df, file.path(out_dir, "01_total_and_domain_scores.csv"), row.names = FALSE)
cat("\n--- Total and domain scores ---\n")
print(scale_df[, c("scale", "n", "pre_M", "post_M", "mean_diff", "t_stat", "df", "p", "dz")], digits = 3, row.names = FALSE)
cat("Saved: output/01_total_and_domain_scores.csv\n")
```

```
# --------------------------------------------------------------------------
# 6. Item-level analysis with Holm-Bonferroni correction across 34 tests
#    Reproduces Table 3 in the manuscript
# --------------------------------------------------------------------------
item_rows <- lapply(ALL_ITEMS, function(i) {
  r        <- paired_stats(dat[[pre_col(i)]], dat[[post_col(i)]], sprintf("Item %d", i))
  r$item    <- i
  r$label   <- ITEM_LABELS[[as.character(i)]]
  r$domain  <- names(DOMAINS)[sapply(DOMAINS, function(x) i %in% x)]
  r
})
item_df <- do.call(rbind, item_rows)
item_df$p_holm    <- p.adjust(item_df$p, method = "holm")
item_df$sig_raw   <- item_df$p     < 0.05
item_df$sig_holm  <- item_df$p_holm < 0.05
item_df <- item_df[, c("item", "label", "domain", "n", "pre_M", "pre_SD",
                  "post_M", "post_SD", "mean_diff", "ci95_low", "ci95_high",
                  "t_stat", "df", "p", "p_holm", "dz", "sig_raw", "sig_holm",
                  "shapiro_p", "wilcoxon_p")]
write.csv(item_df, file.path(out_dir, "02_item_level_analysis.csv"), row.names =
FALSE)
cat(sprintf("\n--- Item-level analysis ---\nSignificant raw p < .05:
%d/34\nSignificant after Holm-Bonferroni: %d/34\n",
          sum(item_df$sig_raw, na.rm = TRUE), sum(item_df$sig_holm, na.rm =
TRUE)))
cat("Saved: output/02_item_level_analysis.csv\n")


# --------------------------------------------------------------------------
# 7. Missingness summary by item
```

```
# --------------------------------------------------------------------------
miss_rows <- lapply(ALL_ITEMS, function(i) {
  data.frame(
    item        = i,
    label       = ITEM_LABELS[[as.character(i)]],
    domain      = names(DOMAINS)[sapply(DOMAINS, function(x) i %in% x)],
    pre_missing = sum(is.na(dat[[pre_col(i)]])),
    post_missing= sum(is.na(dat[[post_col(i)]])),
    valid_pairs = sum(complete.cases(dat[[pre_col(i)]], dat[[post_col(i)]]))
  )
})
miss_df <- do.call(rbind, miss_rows)
write.csv(miss_df, file.path(out_dir, "03_item_missingness.csv"), row.names =
FALSE)
cat("\nSaved: output/03_item_missingness.csv\n")


# --------------------------------------------------------------------------
# 8. Internal consistency: Cronbach's alpha and McDonald's omega
#    (single-factor model via psych::omega, listwise-complete cases)
#    Reproduces α = .783 / ω = .833 (pre) and α = .814 / ω = .893 (post)
#    reported in the manuscript
# --------------------------------------------------------------------------
cat("\n--- Internal consistency (listwise-complete cases on all 34 items) ---\n")
rel_rows <- list()
for (tp in c("pre", "post")) {
  col_fn <- if (tp == "pre") pre_col else post_col
  mat    <- dat[, sapply(ALL_ITEMS, col_fn), drop = FALSE]
  lw     <- mat[complete.cases(mat), ]
```

```r
  cat(sprintf("  %s: %d listwise-complete cases\n", tp, nrow(lw)))


  a  <- suppressWarnings(psych::alpha(lw, warnings = FALSE))
  om <- tryCatch(
    suppressWarnings(psych::omega(lw, nfactors = 1, plot = FALSE, warnings =
FALSE)),
    error = function(e) NULL
  )
  rel_rows[[tp]] <- data.frame(
    timepoint       = tp,
    n_listwise      = nrow(lw),
    cronbach_alpha   = round(a$total$raw_alpha, 3),
    mcdonald_omega   = if (!is.null(om)) round(om$omega.tot, 3) else NA
  )
}
rel_df <- do.call(rbind, rel_rows)
print(rel_df, row.names = FALSE)
write.csv(rel_df, file.path(out_dir, "04_reliability.csv"), row.names = FALSE)
cat("Saved: output/04_reliability.csv\n")


# ---------------------------------------------------------------------------
# 9. Item-rest correlations for items 12, 28, 29 (high-missingness diagnostic)
# ---------------------------------------------------------------------------
item_rest_r <- function(item_no, tp) {
  col_fn <- if (tp == "pre") pre_col else post_col
  dom    <- names(DOMAINS)[sapply(DOMAINS, function(x) item_no %in% x)]
  rest   <- setdiff(DOMAINS[[dom]], item_no)
  iv     <- dat[[col_fn(item_no)]]
```

```r
  rv    <- row_sum_avail(dat[, sapply(rest, col_fn), drop = FALSE])
  ok    <- complete.cases(iv, rv)
  ct    <- cor.test(iv[ok], rv[ok])
  data.frame(item = item_no, timepoint = tp, domain = dom, n = sum(ok),
             item_rest_r = round(unname(ct$estimate), 3), p = ct$p.value)
}
rest_df <- do.call(rbind, lapply(c(12, 28, 29), function(i)
  rbind(item_rest_r(i, "pre"), item_rest_r(i, "post"))))
write.csv(rest_df, file.path(out_dir, "05_item_rest_correlations.csv"), row.names =
FALSE)
cat("\nSaved: output/05_item_rest_correlations.csv\n")


# -------------------------------------------------------------------------
# 10. Subgroup analysis: total-score change by nutritional-status group
#    (Holm-Bonferroni correction across 4 comparisons)
#    Reproduces Table 4 in the manuscript
# -------------------------------------------------------------------------
SUBGROUPS <- c("0" = "No anemia or CCM", "2" = "Anemia only",
               "3" = "CCM only",       "4" = "Anemia + CCM")

sub <- dat[!is.na(dat$nutritional_status_group) &
           as.character(dat$nutritional_status_group) %in% names(SUBGROUPS), ]
sub$subgroup_label <- SUBGROUPS[as.character(sub$nutritional_status_group)]

sub_rows <- lapply(names(SUBGROUPS), function(code) {
  g <- sub[as.character(sub$nutritional_status_group) == code, ]
  paired_stats(g$TOTAL_PRE, g$TOTAL_POST, SUBGROUPS[[code]])
})
```

```
sub_df        <- do.call(rbind, sub_rows)

sub_df$p_holm  <- p.adjust(sub_df$p, method = "holm")

write.csv(sub_df, file.path(out_dir, "06_subgroup_analysis.csv"), row.names =
FALSE)

cat("\n--- Subgroup analysis ---\n")

print(sub_df[, c("scale", "n", "pre_M", "post_M", "mean_diff", "t_stat", "p", "p_holm",
"dz")],

      digits = 3, row.names = FALSE)

cat("Saved: output/06_subgroup_analysis.csv\n")


# --------------------------------------------------------------------------

# 11. Mixed-design ANOVA: subgroup × time interaction on total score

#    Uses afex::aov_ez (Type III SS), reproduces F(3,204) = 2.09, p = .100

# --------------------------------------------------------------------------

sub_c    <- sub[complete.cases(sub$TOTAL_PRE, sub$TOTAL_POST), ]

sub_c$id <- factor(seq_len(nrow(sub_c)))


long <- reshape(

  sub_c[, c("id", "subgroup_label", "TOTAL_PRE", "TOTAL_POST")],

  direction = "long",

  varying   = c("TOTAL_PRE", "TOTAL_POST"),

  v.names   = "score",

  timevar   = "time",

  times     = c("pre", "post")

)

long$time         <- factor(long$time, levels = c("pre", "post"))

long$subgroup_label <- factor(long$subgroup_label)


aov_res <- tryCatch({
```

```r
  afex::aov_ez(id = "id", dv = "score", data = long,
               within = "time", between = "subgroup_label",
               type = 3, anova_table = list(es = "pes"))
}, error = function(e) { cat("[afex failed:", conditionMessage(e), "]\n"); NULL })

if (!is.null(aov_res)) {
  aov_tab      <- as.data.frame(aov_res$anova_table)
  aov_tab$term <- rownames(aov_tab)
  write.csv(aov_tab, file.path(out_dir, "07_mixed_anova.csv"), row.names = FALSE)
  cat("\n--- Mixed ANOVA (subgroup × time) ---\n")
  print(aov_tab, digits = 4)
  cat("Saved: output/07_mixed_anova.csv\n")
}

# ---------------------------------------------------------------------------
# 12. Regression-to-the-mean check
#     Regresses the change score (post – pre) on the baseline total score;
#     a significant negative slope indicates regression to the mean
# ---------------------------------------------------------------------------
rtm       <- dat[complete.cases(dat$TOTAL_PRE, dat$TOTAL_POST), ]
rtm$delta <- rtm$TOTAL_POST - rtm$TOTAL_PRE
rtm_mod   <- lm(delta ~ TOTAL_PRE, data = rtm)
cat("\n--- Regression-to-the-mean check ---\n")
print(summary(rtm_mod))
sink(file.path(out_dir, "08_rtm_check.txt"))
print(summary(rtm_mod))
sink()
cat("Saved: output/08_rtm_check.txt\n")
```

```
# ------------------------------------------------------------------------
# 13. Sample descriptives
# ------------------------------------------------------------------------
cat(sprintf("\n--- Sample descriptives ---\nn = %d\nMaternal age: M = %.2f, SD = %.2f, range = %d–%d\n",
        nrow(dat),
        mean(dat$maternal_age_years, na.rm = TRUE),
        sd(dat$maternal_age_years,   na.rm = TRUE),
        min(dat$maternal_age_years,  na.rm = TRUE),
        max(dat$maternal_age_years,  na.rm = TRUE)))
cat(sprintf("Participants under 18: n = %d\n",
        sum(dat$maternal_age_years < 18, na.rm = TRUE)))

# ------------------------------------------------------------------------
# 14. Consolidated Excel workbook
# ------------------------------------------------------------------------
wb <- createWorkbook()
sheet_list <- list(
  "Scores_domains"    = scale_df,
  "Items_Holm"        = item_df,
  "Missingness"       = miss_df,
  "Reliability"       = rel_df,
  "Item_rest_corr"    = rest_df,
  "Subgroups"         = sub_df
)
if (!is.null(aov_res)) sheet_list[["ANOVA_group_x_time"]] <- aov_tab
```

```
for (sname in names(sheet_list)) {
  addWorksheet(wb, sname)
  writeData(wb, sname, sheet_list[[sname]])
}
xlsx_path <- file.path(out_dir, "EPC-DCI_results.xlsx")
saveWorkbook(wb, xlsx_path, overwrite = TRUE)
cat(sprintf("\nConsolidated workbook saved: %s\n", xlsx_path))
cat("\nAll analyses complete.\n")
```
