# Supplementary material for: Changes in family knowledge, practices, and perceived competencies after a family-centered home-visiting program for chronic child malnutrition prevention in rural Ecuador: an uncontrolled pre-post study
Source: Front Public Health. 2026 Oct 26;14:1925365. doi: 10.3389/fpubh.2026.1925365 (PMC13745657; doi:10.3389/fpubh.2026.1925365)
Supplement: Supplementary file 2 [file Supplementary_file_1.DOCX]

**Supplementary Appendix**

**EPC-DCI Instrument: Scoring Guide and Domain Composition**

**Changes in Family Knowledge, Practices, and Perceived Competencies After a Family-Centered Home-Visiting Program for Chronic Child Malnutrition Prevention in Rural Ecuador**

**1. Instrument Overview**

The Encuesta de Prácticas Familiares en Desnutrición Crónica Infantil (EPC-DCI) is a 34-item scale developed for this study to assess parental knowledge, practices, and perceived competencies related to the prevention and management of chronic child malnutrition (CCM). All items use a 6-point Likert-type response scale anchored at 1 (Strongly disagree) and 6 (Strongly agree).

No items are reverse-scored. The instrument was administered at baseline (Pre-FCP) and at the end of the six-month home-visiting programme (Post-FCP).

The scale is organised into three content domains that reflect the primary targets of the Family-Centered Practices (FCP) intervention:

- Knowledge (10 items): items assessing awareness and understanding of nutritional needs, breastfeeding, and CCM prevention.
- Practices (12 items): items assessing self-reported feeding, dietary, hygiene, and healthcare behaviours.
- Competencies (12 items): items assessing perceived self-efficacy and confidence in family nutrition and childcare roles.

Domain scores are calculated as the sum of all non-missing items within each domain (available-case scoring). The total scale score is the sum of all 34 item responses with at least one non-missing item per participant. Theoretical ranges are: Knowledge 10–60, Practices 12–72, Competencies 12–72, Total scale 34–204.

**2. Items by Domain**

The table below lists all 34 items in their assigned domain, the original item number (as printed on the questionnaire and used in the data file), the full English wording, the scoring direction, and the item-level response format.

| **Item #** | **Domain** | **Item wording** | **Scoring** | **Notes** |
| --- | --- | --- | --- | --- |
| Knowledge domain (10 items) α Pre = .76, Post = .82 ω Pre = .79, Post = .83 Theoretical range: 10–60 | | | | |
| 6 | Knowledge | I need more knowledge to improve my family's nutrition. | Direct (1–6) | — |
| 13 | Knowledge | I received adequate information about breastfeeding. | Direct (1–6) | — |
| 15 | Knowledge | I know the benefits of continuing breastfeeding for two years. | Direct (1–6) | — |
| 23 | Knowledge | I have the knowledge to take good care of my children. | Direct (1–6) | — |
| 25 | Knowledge | I believe I need to learn more about children's overall development and health. | Direct (1–6) | — |
| 28 | Knowledge | I know what to do if my child has experienced or experiences stunted growth. [wording corrected] | Direct (1–6) | High missingness; Wording corrected |
| 29 | Knowledge | I know what to do if my child has experienced or experiences low weight. [wording corrected] | Direct (1–6) | High missingness; Wording corrected |
| 32 | Knowledge | We know the problems our child can have in case of CCM. | Direct (1–6) | — |
| 33 | Knowledge | We know how to prevent chronic malnutrition. | Direct (1–6) | — |
| 34 | Knowledge | I have the necessary information about what to do if our child experiences CCM. | Direct (1–6) | — |
| Practices domain (12 items) α Pre = .78, Post = .90 ω Pre = .80, Post = .91 Theoretical range: 12–72 | | | | |
| 1 | Practices | Our family has a stable and adequate food supply. | Direct (1–6) | — |
| 2 | Practices | We regularly share a daily meal together. | Direct (1–6) | — |
| 5 | Practices | My daily meals include a variety of foods. | Direct (1–6) | — |
| 7 | Practices | As a mother, I eat a healthy and adequate diet. | Direct (1–6) | — |
| 8 | Practices | As a mother, my daily diet includes a variety of foods. | Direct (1–6) | — |
| 10 | Practices | I pay attention to my children's hygiene during meals. | Direct (1–6) | — |
| 11 | Practices | During my pregnancy, I had a nutritious and healthy diet. | Direct (1–6) | — |
| 12 | Practices | I know the recommendations for introducing complementary foods safely and nutritiously. | Direct (1–6) | High missingness |
| 14 | Practices | My child was breastfed for more than six months. | Direct (1–6) | — |
| 16 | Practices | Starting at 6 months, my child received complementary foods. | Direct (1–6) | — |
| 20 | Practices | My child has regular access to medical checkups. | Direct (1–6) | — |
| 24 | Practices | I practice proper food hygiene to prevent illness. | Direct (1–6) | — |
| Competencies domain (12 items) α Pre = .82, Post = .92 ω Pre = .83, Post = .92 Theoretical range: 12–72 | | | | |
| 3 | Competencies | I am satisfied with the food I serve at the table. | Direct (1–6) | — |
| 4 | Competencies | I feel that I feed my family properly. | Direct (1–6) | — |
| 9 | Competencies | I know what to do when my child displays challenging behaviors during mealtimes. | Direct (1–6) | — |
| 17 | Competencies | I have had a positive breastfeeding experience. | Direct (1–6) | — |
| 18 | Competencies | I know how to provide healthy nutrition for my child. | Direct (1–6) | — |
| 19 | Competencies | I can proactively manage my own health. | Direct (1–6) | — |
| 21 | Competencies | My family enjoys good health. | Direct (1–6) | — |
| 22 | Competencies | My child is currently in good health. | Direct (1–6) | — |
| 26 | Competencies | I make sure my child gets all recommended vaccinations. | Direct (1–6) | — |
| 27 | Competencies | As a mother, I make sure my child attends medical appointments for growth/weight monitoring. | Direct (1–6) | — |
| 30 | Competencies | I did everything necessary to stay healthy during my pregnancy. | Direct (1–6) | — |
| 31 | Competencies | Our family receives support from organizations promoting child well-being. | Direct (1–6) | — |

**3. Scoring Procedure**

Response scale. Each item is rated on a 6-point Likert scale: 1 = Strongly disagree, 2 = Disagree, 3 = Somewhat disagree, 4 = Somewhat agree, 5 = Agree, 6 = Strongly agree.

Domain scores. The domain score is the unweighted sum of the available responses within that domain. No items are reverse-scored. A participant's domain score is retained as long as at least one item in that domain was answered (available-case scoring); participants with all items in a domain missing receive NA for that domain.

Total scale score. The total score is the sum of all 34 item responses. As with domain scores, available-case scoring is used.

Missing data. Items 12 (complementary feeding), 28 (stunted growth), and 29 (low weight) showed substantially higher missingness than the remaining 31 items, because these items were only applicable to a subset of participants (e.g., mothers of infants younger than six months, or families with a child formally identified with CCM). Valid-pair sample sizes for these three items were n = 71, 84, and 61, respectively.

**4. Item-Rest Correlations and Internal Consistency**

Total-scale internal consistency (all 34 items, listwise-complete cases) was α = .783 (ω = .833) at Pre-FCP and α = .814 (ω = .893) at Post-FCP. McDonald's ω was estimated via a single-factor model using the psych package in R (Revelle, 2024). Domain-level values (pairwise-available cases) are shown in the domain sub-headers of the table above.

Items 12, 28, and 29 showed positive and statistically significant item-rest correlations at both time points, supporting their retention in the scale despite high missingness. Their corrected item-total correlations ranged from .30 to .58 across domains and time points.
